# Soil conditioners strategies for enhancing selenium bioavailability, and maize biomass in selenium-rich dryland soils

**DOI:** 10.3389/fpls.2026.1776194

**Published:** 2026-03-04

**Authors:** Taiqing Huang, Shuyi Liu, Manling Liao, Jianhua Wang, Xiaohui Peng, Chunxiang Wei, Yanfei Huang, Zhong Liu, Bin Liu, Qizhan Tang, Zepu Jiang

**Affiliations:** 1 Agricultural Resource and Environment Research Institute, Guangxi Academy of Agricultural Sciences, Nanning, China; 2 Guangxi Academy of Agricultural Sciences, Nanning, China; 3 Soil Fertilizer and Ecological Station of Hezhou City, Hezhou, Guangxi, China; 4 Hechi Agricultural Science Research Institute, Hechi, Guangxi, China

**Keywords:** maize, selenium content, soil conditioner, soil selenium speciation, transfer coefficient

## Abstract

Low selenium bioavailability in selenium-rich soils represents a key constraint limiting selenium biofortification in agriculture. This study evaluated soil conditioning strategies to enhance selenium bioavailability, providing a theoretical foundation for efficient utilization of selenium-enriched soil resources. A pot experiment tested four soil conditioners: organic fertilizer, potassium humate, lime, and biochar, across three consecutive maize plantings. Soil conditioners effectively modified soil physicochemical properties: lime significantly increased pH and available phosphorus, while organic fertilizer increased available sulfur. These amendments markedly affected soluble and exchangeable selenium fractions. All treatments progressively increased the proportion of soluble selenium, with lime and biochar showing the most substantial gains in batches two and three (0.59%, 0.23%, 0.67%, and 0.30%, respectively). Organic fertilizer, lime, and biochar consistently elevated root selenium concentrations across all three batches by 7.79–11.01%, 33.31–135.41%, and 28.84–40.81%, respectively. Potassium humate increased root selenium by 9.04–26.42% in batches two and three. Notably, only lime consistently enhanced shoot selenium by 40.13–87.38% across all batches, while biochar increased shoot selenium by 5.60% in batch three. Plant selenium translocation analysis revealed that only lime treatment in batch three significantly increased the selenium transfer coefficient. Correlation analysis demonstrated a highly significant positive relationship between shoot selenium content and soil pH, whereas root selenium showed no significant correlation with soluble or exchangeable selenium fractions. In selenium-rich dryland soils, conditioner application increases soil pH, thereby enhancing selenium availability and root absorption. Lime proved most effective for increasing crop selenium content, while biochar also substantially improved soil selenium availability.

## 1 Introduction

Selenium is an essential micronutrient for human health (Cao et al., 2025). Plant-derived selenium serves as the primary dietary source for humans (Rayman, 2008; Gu et al., 2025). Globally, approximately 1 billion people experience inadequate daily selenium intake (Haug et al., 2007; Gu et al., 2025), with this proportion reaching 36%–61% in China (Dinh et al., 2018; Cao et al., 2025). Soil selenium represents the main source of plant selenium accumulation. Plant selenium content depends on the total selenium content of selenium-rich soil (Wu et al., 2025). Selenium undergoes interconversion among various chemical forms in soil through oxidation-reduction, humification, and methylation (Pu et al., 2023). Soil environmental conditions significantly influence selenium speciation, with higher available selenium content promoting selenium absorption and accumulation in crops (Dinh et al., 2019). Although total selenium content in southern China soils is generally high (Zhao et al., 2024), selenium levels in many agricultural products remain low (Du et al., 2018). Excessive selenium (Se) acts as a pro-oxidant in plants, causing significant toxicity (selenosis) characterized by stunted growth, root inhibition, chlorosis, and necrosis (Gu et al., 2025). It causes damage by replacing sulfur in amino acids (cysteine and methionine), resulting in dysfunctional proteins, alongside inducing severe oxidative stress (Cao et al., 2025). By modulating soil physical and chemical properties to improve selenium bioavailability, crop selenium absorption and accumulation can be enhanced, thereby improving human selenium nutrition status.

Plant roots primarily absorb water-soluble chemical selenium forms such as Se^6+^, Se^4+^, and organic selenium from soil solution (White, 2018). These water-soluble chemical selenium forms exist predominantly as water-soluble and partially exchangeable fractions, collectively termed extractable selenium (Loffredo et al., 2011). Studies of typical selenium-rich soils in China indicate that these two fractions account for approximately 0.7% and 5.1% of total selenium in the soil, respectively (Lyu et al., 2021). Soil pH, mineral composition, and organic matter content are closely related to soil selenium speciation characteristics (Winkel et al., 2015). Selenate (SeO_4_^2–^) is the main water-soluble form of Se in oxic soils (pH + pe > 15), which include most cultivated soils, whereas selenite (SeO_3_^2–^) predominates in anaerobic soils with a neutral to acidic pH (pH + pe = 7.5–15), such as paddy soils (Fordyce, 2013; Pilbeam et al., 2015). Selenide (Se^2–^) species are stable only under low redox conditions (pH + pe < 7.5) and are rarely present in cultivated soils. Selenate is relatively mobile in the soil solution, but selenite is strongly absorbed by iron and aluminum oxides/hydroxides and, to a lesser extent, by clays and organic matter (Pilbeam et al., 2015). Thus, the addition of selenate to soils facilitates immediate Se accumulation by plants, while selenite provides a longer lasting Se fertilizer (Fordyce, 2013; Pilbeam et al., 2015). Soil organic matter and iron oxides both adsorb selenium, reducing its bioavailability (Qin et al., 2023).

Adding soil conditioners can modify soil physicochemical properties, thereby influencing the cycling and transformation of soil elements (Huang et al., 2024). Biochar application in paddy fields reduces the selenium uptake by rice roots (Huang et al., 2019), whereas in dryland soils, it enhances plant selenium absorption (Ma et al., 2023). Organic fertilizer is often reported to decrease selenium bioavailability by adsorbing and immobilizing soil selenium (Li et al., 2017; Sharma et al., 2011); however, long-term field experiments show that wheat grains grown with organic fertilizer contain significantly higher selenium than those receiving chemical fertilizer (Daud et al., 2024). Returning selenium-enriched crop straw to the field can also increase selenium content in subsequent crops (Wang et al., 2018). Yet conventional straw return promotes the transformation of water-soluble small-molecule organic selenium (FA-Se) into large-molecule humic-bound selenium (HA-Se), reducing soil selenium bioavailability (Wang et al., 2020).

Studies using selenium-enriched soils with added exogenous selenium demonstrate that sulfur application significantly decreases water-soluble selenium while promoting its association with iron and manganese oxides and organic matter, thereby reducing plant selenium content (Deng et al., 2021). In contrast, in naturally selenium-poor soils without exogenous selenium, sulfur application can enhance wheat selenium content (Yeasmin et al., 2022). Dinh et al. (2019) showed that lime and coal slag can regulate selenium availability in acidic selenium-rich soils in southern China, but the effects of the same amendments vary by soil type, either increasing or decreasing available selenium (Liu et al., 2021). Xie et al. (2017) studied the effects of phosphorus application on soil selenium speciation and wheat plant selenium content, showing that different phosphorus sources had different effects. The application of urea phosphate and ammonium polyphosphate did not significantly alter soil selenium fractions or wheat selenium content in different organs (An et al., 2019). In contrast, lime and disodium hydrogen phosphate increased water-soluble and exchangeable selenium and decreased residual selenium, effectively improving selenium availability and uptake in rice (Tan et al., 2024).

Overall, the impact of soil amendments on selenium bioavailability is inconsistent, reflecting the strong influence of local soil conditions on the effectiveness of selenium-activating agricultural measures (Dinh et al., 2019). These discrepancies may arise from regional differences in the dominant factors controlling soil selenium bioavailability (Farooq et al., 2023). In southern China, soils are generally high in total selenium but low in available selenium, yet studies on selenium activation remain limited. This study focuses on typical selenium-rich red soils in this region, using maize as a representative crop, to evaluate the effects of different soil conditioners on selenium speciation transformation and bioavailability in dryland soils, providing a theoretical foundation and practical guidance for the efficient utilization of selenium-rich soils in this area.

## 2 Materials and methods

### 2.1 Sample collection

The experiment was conducted in a greenhouse at the Guangxi Academy of Agricultural Sciences Research and Experiment Base from March to August 2023. Potting soil was collected from Yandong Township, Debao County, Baise City, Guangxi Province. Its basic physicochemical properties were: organic matter 30.3 g/kg, total nitrogen 1.8 g/kg, total phosphorus 1.25 g/kg, total potassium 29.22 g/kg, available nitrogen 92 mg/kg, available phosphorus 77 mg/kg, available potassium 183 mg/kg, pH 7.0, and total selenium 0.82 mg/kg (**Table 1**).

**Table 1:** Soil basic physicochemical properties.

pH	OM (g/kg)	TN (g/kg)	TP (g/kg)	TK (g/kg)	TSe (mg/kg)	AN (mg/kg)	AP (mg/kg)	AK (mg/kg)
7.00	30.30	1.80	1.25	29.22	0.82	92.00	77.00	183.00

OM, organic matter; TN, total nitrogen; TP, total phosphorus; TK, total potassium; TSe, total selenium; AN, available nitrogen; AP, available phosphorus; AK, available potassium.

The soil conditioners used were as follows: organic fertilizer containing 461 g/kg organic matter, N 29 g/kg, P_2O5_ 11 g/kg, K_2_O 19 g/kg, pH 5.5; potassium humate with 373 g/kg organic matter, N 24.1 g/kg, P_205_ 6 g/kg, K_2_O 61.2 g/kg; and biochar with 423 g/kg organic C, total nitrogen 11.1 g/kg, P_205_ 7 g/kg, K_2_O 32.6 g/kg, pH 9.3. The maize variety used was Guidan 162.

### 2.2 Experimental design

The experiment was performed in pots with five treatments: organic fertilizer (OF, 3.13 g/kg soil), quicklime (QL, 1.25 g/kg soil), biochar (BC, 10 g/kg soil), potassium humate (FA, 5 g/kg soil), and a conventional fertilization control (CK). Chemical fertilizers were applied at the same rate across all treatments. Each treatment was replicated four times. All soil conditioners were thoroughly mixed with the soil and then placed in the pots before planting the first batch of corn. No soil conditioners were added for the second and third batches of corn planted thereafter.

Biochar was thoroughly mixed with the soil before planting the first maize batch; no biochar was added for subsequent batches. Each batch received only basal fertilizer (compound fertilizer 15-15-15, 3.00 g/pot), mixed with the soil prior to planting. Maize seeds were sown at five per pot, thinned to two plants per pot at the three-leaf stage. Irrigation was applied daily to maintain 70% of field capacity. Three consecutive maize batches were planted, each grown for 30 days.

### 2.3 Sample collection and analysis

#### 2.3.1 Sample collection and preparation

After each 30-day growth period, maize roots, stems, and leaves were harvested, washed, dried, weighed, and ground for total selenium analysis. Simultaneously, pot soils were thoroughly mixed and sampled for physicochemical property assessment and selenium speciation analysis.

#### 2.3.2 Analytical methods

Soil pH was measured potentiometrically (soil-water ratio 1:2.5). Available phosphorus was determined via sodium carbonate extraction-molybdenum antimony colorimetry, and available sulfur via phosphate extraction-barium sulfate turbidimetry. Soil total selenium was quantified using hydride generation-atomic fluorescence spectrometry according to NY/T 1104-2006 (Cao et al., 2025).

Plant total selenium was determined following GB 5009.93-2017. Briefly, 0.3000 g of plant material was digested in 8 mL concentrated nitric acid at 120 °C for 30 min, followed by microwave digestion (800 W for 10 min, hold 5 min; 1400 W for 10 min, hold 20 min) (An et al., 2019). The digest was cooled, reduced to 2 mL, treated with 5 mL 1:1 HCl, digested for 15 min, diluted to 50 mL, and analyzed using an atomic fluorescence spectrometer (Jitian AFS-8330).

Soil selenium speciation was determined using a Deng et al. (2021) five-step sequential extraction method. One gram of air-dried, sieved soil (0.15 mm) was extracted at a 1:10 soil-to-solution ratio: (i) soluble Se (Sol-Se) with 0.25 mol/L KCl at 25 °C for 1 h; (ii) exchangeable Se (Exc-Se) with 0.7 mol/L KH_2_PO_4_ (pH 5.0) at 25 °C for 4 h; (iii) iron/manganese oxide-bound Se (FMO-Se) with 2.5 mol/L HCl at 90 °C for 4 h; (iv) organic matter-bound Se (OM-Se) with 8 mL 5% K_2_S_2_O_8_ + 2 mL 1:1 HNO_3_ at 90°C for 3 h; (v) residual Se (Res-Se) determined on the remaining soil using NY/T 1104-2006. Supernatants were centrifuged at 4000 rpm for 10 min and stored at 4 °C for analysis (Deng et al., 2021).

### 2.4 Data analysis

The data were organized using Microsoft Excel 2010 (Microsoft Corp., Raymond, Washington, USA) and SPSS version 26. ANOVA was employed to analyze the effects of differences between treatments were assessed by least significant difference (LSD) tests (*P <* 0.05). Figures, correlation analysis and plotting were conducted using Origin 2021 (Origin Lab, Corp., Hampton, Massachusetts, USA).

## 3 Results

### 3.1 Effects of soil conditioner application on soil pH, available phosphorus, and available sulfur

**Figure 1:**
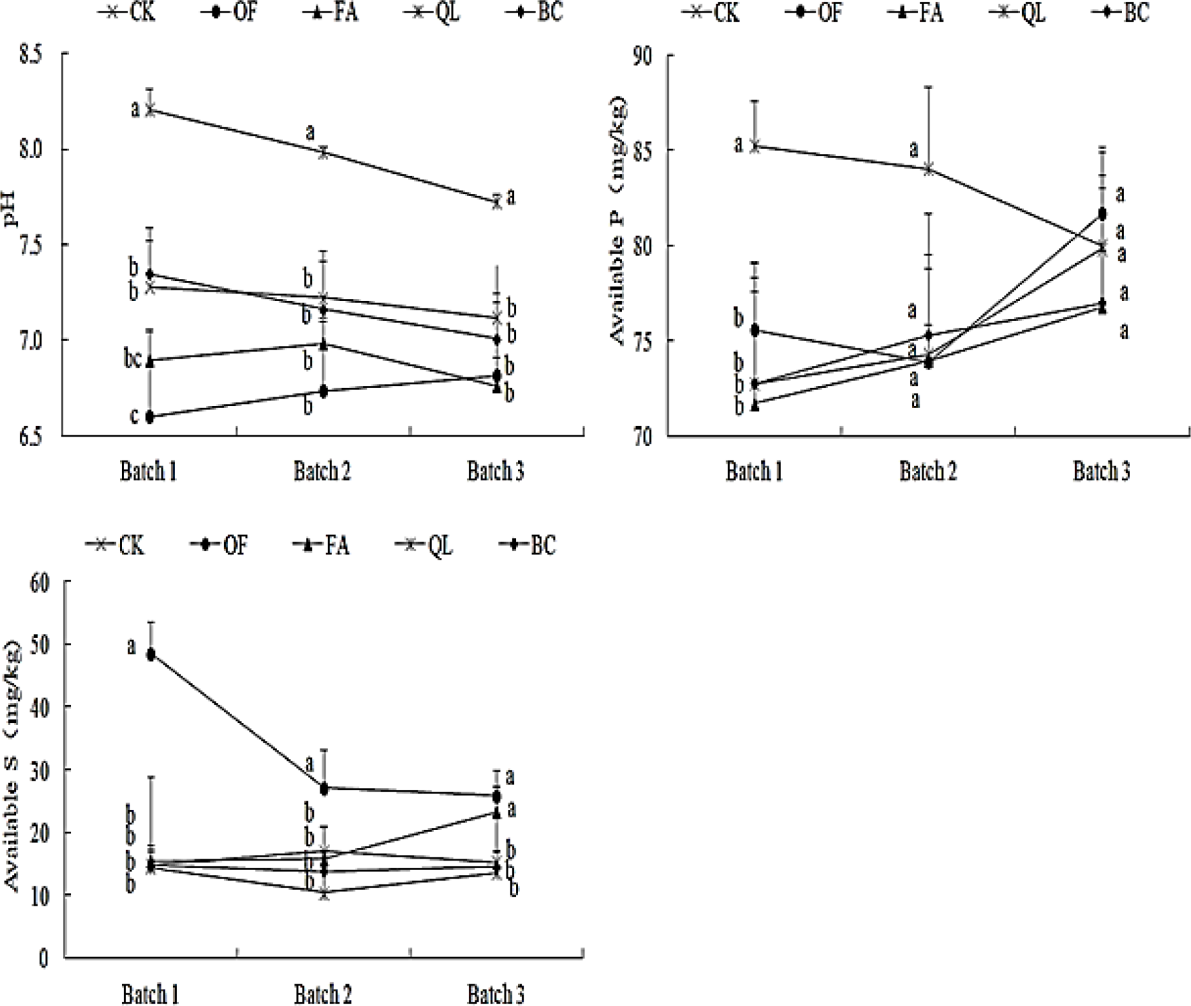
Variation of soil pH, available phosphorus, and available sulfur under different soil conditioner treatments. Different lowercase letters within each batch indicate significant differences among treatments (*P* < 0.05). The vertical bars represent the standard error of the mean (n = 4).

**Figure 1** illustrates the dynamics of these parameters under different soil conditioner treatments. Soil pH under lime, biochar, and conventional treatments initially increased and then gradually declined. In contrast, potassium humate treatment exhibited a decrease followed by an increase and a subsequent decline, while organic fertilizer initially reduced pH, which then gradually increased. Among all treatments, lime consistently maintained the highest pH. Except for the first maize batch under organic fertilizer, which showed significantly lower pH than biochar and conventional treatments, there were no significant pH differences among biochar, conventional, potassium humate, and organic fertilizer treatments after the second and third maize batches. Overall, pH remained higher under biochar and conventional treatments compared to potassium humate and organic fertilizer.

Available phosphorus levels were significantly higher only under the conventional treatment during the first two maize batches, while no significant differences were observed among lime, biochar, potassium humate, and organic fertilizer treatments. After the third batch, available phosphorus levels showed no significant differences among any treatments.

Available sulfur content was significantly higher in soils treated with organic fertilizer after the first and second maize batches, whereas other treatments showed no significant differences. In the third batch, available sulfur increased under potassium humate treatment, resulting in both organic fertilizer and potassium humate treatments having significantly higher sulfur content than the other three treatments. No significant differences were observed between organic fertilizer and potassium humate, or among lime, biochar, and conventional treatments.

### 3.2 Effects of soil conditioning on soil selenium speciation

**Table 2** presents the proportions of soil selenium forms following application of different soil conditioners. After the first maize batch, soil conditioners primarily influenced exchangeable and residual selenium, whereas soluble selenium, iron-manganese-bound selenium, and organically bound selenium showed no significant differences among treatments. Exchangeable selenium was highest under organic fertilizer, significantly exceeding all other treatments, and lowest under the control (CK). Residual selenium was highest in the biochar treatment, surpassing lime, potassium humate, organic fertilizer, and CK by 3.91, 5.03, 5.78, and 2.97 percentage points, respectively. Available selenium proportions in organic fertilizer, potassium humate, lime, and biochar treatments were 3.79%, 2.38%, 2.56%, and 2.23%, respectively, all higher than CK.

**Table 2:** Soil selenium speciation composition under different soil conditioner treatments.

Treatments		Soluble-Se (%)	Exchangeable-Se (%)	Fe/Mn oxide-bound-Se (%)	Organic matter-bond-Se (%)	Residual-Se (%)
Batch 1	CK	0.44 ± 0.04 a	1.23 ± 0.35 c	46.90 ± 2.95 a	30.93 ± 0.60 a	20.50 ± 3.18 ab
	OF	0.45 ± 0.03 a	3.34 ± 0.28 a	45.69 ± 0.87 a	32.83 ± 0.22 a	17.68 ± 0.85 b
	FA	0.52 ± 0.10 a	1.86 ± 0.30 bc	45.84 ± 1.40 a	33.34 ± 1.16 a	18.44 ± 1.89 b
	QL	0.51 ± 0.07 a	2.05 ± 0.46 b	45.48 ± 2.38 a	32.40 ± 2.03 a	19.56 ± 1.91 ab
	BC	0.51 ± 0.07 a	1.72 ± 0.26 bc	43.20 ± 2.09 a	31.10 ± 0.97 a	23.47 ± 2.27 a
Batch 2	CK	0.36 ± 0.05 b	2.36 ± 0.23 b	40.30 ± 4.98 a	32.84 ± 5.88 ab	24.13 ± 4.41 a
	OF	0.36 ± 0.06 b	0.21 ± 0.05 c	36.28 ± 2.21 a	38.90 ± 0.82 a	24.26 ± 2.37 a
	FA	0.41 ± 0.07 b	0.38 ± 0.07 c	38.87 ± 3.48 a	36.56 ± 3.49 ab	23.78 ± 1.48 a
	QL	0.95 ± 0.25 a	0.77 ± 0.41 c	41.81 ± 1.56 a	34.18 ± 1.56 ab	22.29 ± 1.87 a
	BC	1.03 ± 0.11 a	5.51 ± 1.35 a	40.05 ± 1.35 a	30.74 ± 1.36 b	22.68 ± 2.54 a
Batch 3	CK	0.12 ± 0.06 b	2.76 ± 0.43 bc	32.99 ± 1.84 a	28.85 ± 0.93 a	35.28 ± 1.56 ab
	OF	0.20 ± 0.05 b	2.11 ± 0.42 c	34.62 ± 2.95 a	29.50 ± 0.39 a	33.57 ± 2.37 ab
	FA	0.40 ± 0.06 a	3.73 ± 0.54 b	30.75 ± 1.80 a	29.42 ± 1.41 a	35.70 ± 0.61 a
	QL	0.35 ± 0.08 a	5.34 ± 0.72 a	32.01 ± 1.85 a	29.66 ± 3.25a	32.64 ± 2.18 ab
	BC	0.41 ± 0.04 a	3.20 ± 0.08 b	32.09 ± 2.93 a	32.41 ± 2.58 a	31.88 ± 0.93 b

Different lowercase letters within the same batch and selenium form indicate significant differences among treatments (*P* < 0.05).

After the second maize harvest, soil conditioners significantly affected soluble, exchangeable, and organically bound selenium, while iron-manganese-bound and residual selenium remained unaffected (**Table 2**). Biochar and lime significantly increased soluble selenium, whereas potassium humate and organic fertilizer treatments were comparable to CK. Exchangeable selenium increased significantly in the biochar treatment, but decreased under lime, potassium humate, and organic fertilizer compared to CK. Organically bound selenium did not differ significantly from CK, though organic fertilizer treatment exceeded biochar. Available selenium decreased in organic fertilizer, potassium humate, and lime treatments but increased in the biochar treatment relative to CK.

After the third maize batch, soil conditioners significantly influenced soluble, exchangeable, and residual selenium, but not iron-manganese-bound or organically bound selenium (**Table 2**). Biochar, lime, and potassium humate increased soluble selenium; lime also enhanced exchangeable selenium, while biochar and potassium humate increased it by 2.14 and 1.61 percentage points, respectively. Exchangeable and soluble selenium under organic fertilizer did not differ from CK. Residual selenium was significantly higher in potassium humate than in biochar, while CK values were comparable across treatments. Available selenium decreased by 0.57 percentage points under organic fertilizer, but increased by 1.25, 2.81, and 0.74 percentage points under potassium humate, lime, and biochar, respectively.

Overall, soil selenium continuously transforms among its forms. Iron-manganese-bound and residual selenium vary significantly between batches, reflecting dynamic soil-environment interactions. Despite these transformations, soluble and exchangeable selenium, which are directly available to crops, remain consistently low.

### 3.3 Maize growth characteristics under different soil conditioning measures

**Figure 2:**
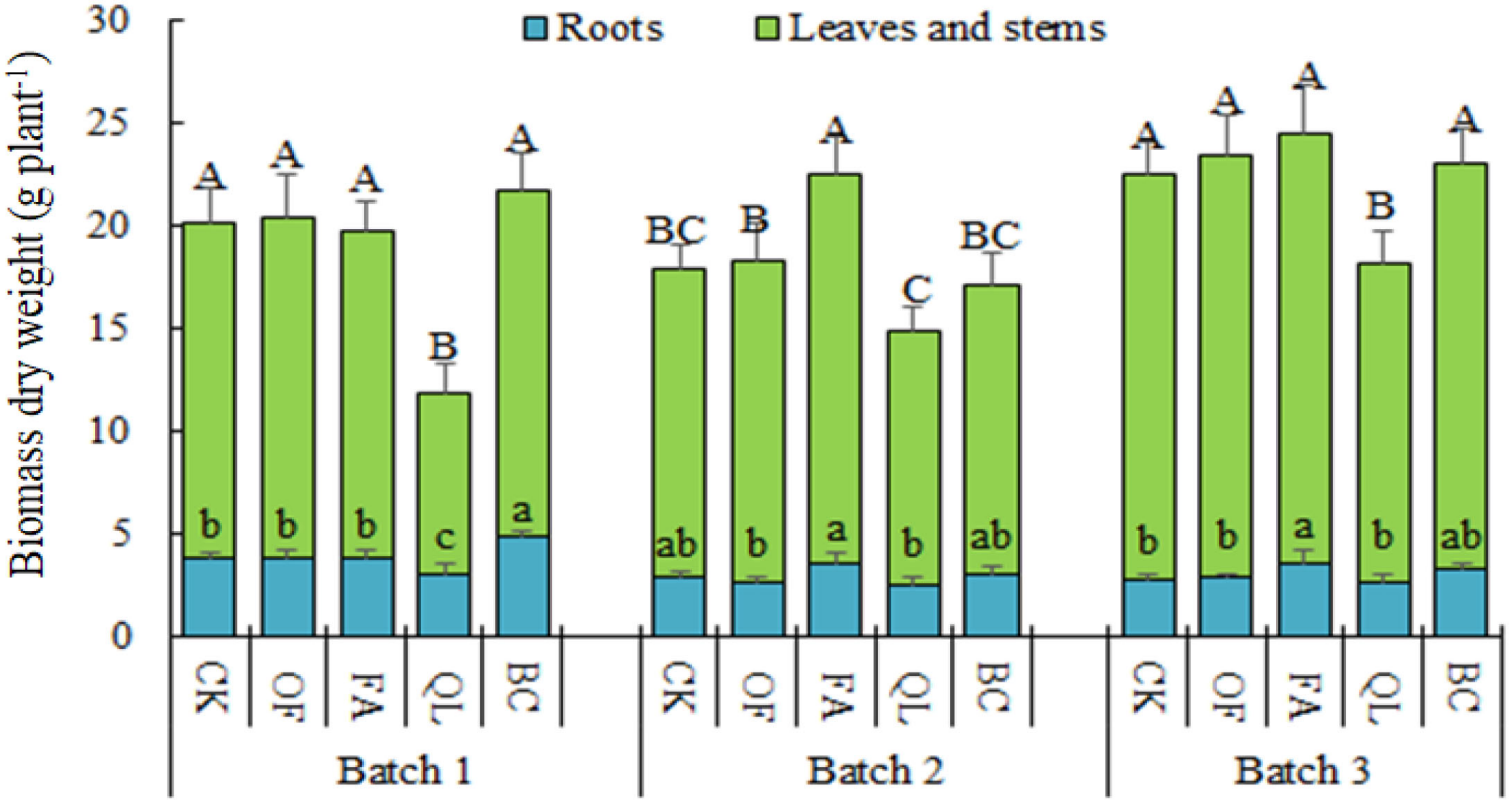
Biomass of consecutively planted maize under different soil conditioner treatments. Different lowercase letters within the same batch indicate significant differences in root biomass among treatments (*P* < 0.05); different uppercase letters indicate significant differences in stem and leaf biomass among treatments (*P* < 0.05).

Total biomass of the first maize batch followed the order: biochar > organic fertilizer > CK > potassium humate > lime (**Figure 2**). Compared to CK, biochar increased total biomass by 7.89%, whereas lime reduced it by 41.17%. Biochar notably enhanced root biomass by 30.20%, while lime reduced root biomass by 18.54%. Stem and leaf biomass did not differ significantly among biochar, potassium humate, organic fertilizer, and CK; however, lime significantly decreased stem and leaf biomass by 46.39%.

After the second maize batch, total biomass ranked as potassium humate > organic fertilizer > CK > biochar > lime (**Figure 2**). Root biomass did not differ significantly among most treatments and the control, although potassium humate roots were significantly heavier than those under biochar and lime. Stem and leaf biomass was highest under potassium humate, increasing 25.85% compared to CK, whereas lime reduced stem and leaf biomass by 18.29%. Organic fertilizer and biochar had no significant effect on stem and leaf biomass relative to CK.

Following the third maize batch, total biomass followed the order: potassium humate > organic fertilizer > biochar > CK > lime (**Figure 2**). Total biomass increased by 9.04%, 4.42%, and 2.54% under potassium humate, organic fertilizer, and biochar, respectively, relative to CK, whereas lime reduced total biomass by 19.42%. Potassium humate significantly enhanced root biomass by 29.53%, while other conditioners showed no significant difference compared to CK. Lime treatment caused a 21.42% reduction in stem and leaf biomass, whereas the other treatments were slightly higher than CK but not significantly different. Across the three maize batches, lime consistently inhibited growth. Organic fertilizers, potassium humate, and biochar exerted modest growth-promoting effects that became more apparent across consecutive plantings, with potassium humate showing the strongest positive impact on maize biomass.

### 3.4 Effects of soil conditioner applications on maize selenium nutrition

#### 3.4.1 Effects of soil conditioners on selenium content in maize

**Figure 3:**
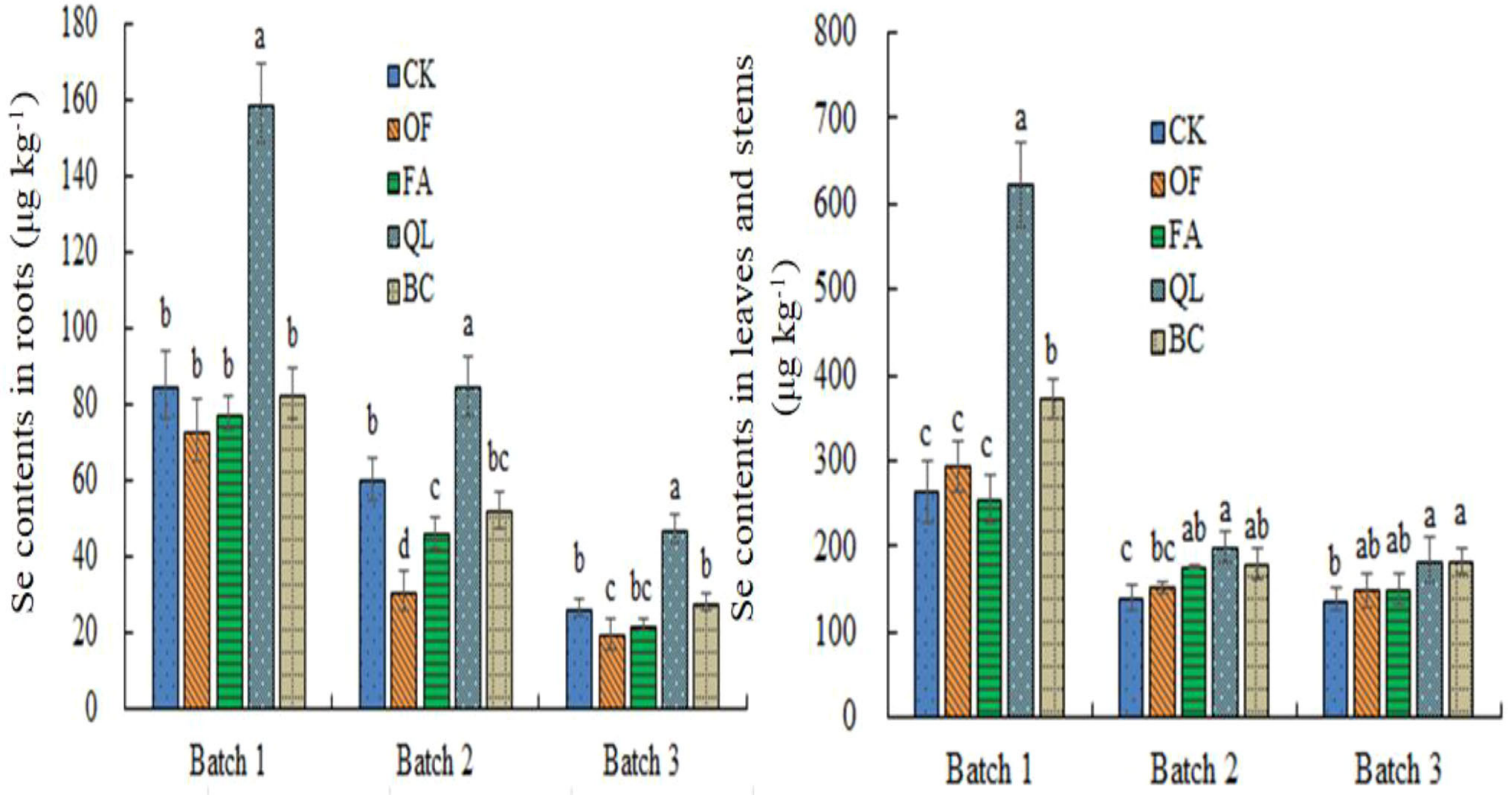
Selenium content in maize roots and stems/leaves under different soil conditioning treatments. Different lowercase letters within the same batch column indicate significant differences among treatments (*P* < 0.05).

Soil conditioners altered selenium uptake and partitioning in maize, with distinct effects on roots versus aboveground tissues (**Figure 3**). In roots, selenium content in nearly all conditioner treatments—across all batches—was higher than in the conventional treatment (CK), except for the first batch under potassium humate (**Figure 3**). Lime and biochar significantly increased root selenium content relative to CK, and both were significantly higher than the organic fertilizer and potassium humate treatments; lime was also significantly higher than biochar. No significant differences were observed among CK, organic fertilizer, and potassium humate.

In stems and leaves, selenium content was generally lower under soil conditioner treatments than CK, except for the lime treatment and the third batch under biochar (**Figure 3**). Selenium content in stems and leaves under lime increased by 87.38%, 40.13%, and 77.71% across the first to third batches, respectively, relative to CK. In contrast, organic fertilizer lowered selenium content by 13.67%, 48.85%, and 25.31% across the three batches, with significant decreases in the latter two. Potassium humate reduced stem and leaf selenium content by 8.48%, 23.15%, and 16.78% across the three batches, with a significant decrease in the second batch. Biochar reduced selenium content by 2.31% and 13.88% in the first two batches, but produced a slight (non-significant) increase of 5.60% in the third batch.

These findings indicate that while several soil conditioners enhance selenium absorption by roots, translocation of root-absorbed selenium to aboveground tissues remains limited. Differences in activation dynamics among conditioners are also evident, with biochar likely requiring longer periods to exert its effects.

#### 3.4.2 Effects of conditioners on selenium accumulation in maize

**Figure 4:**
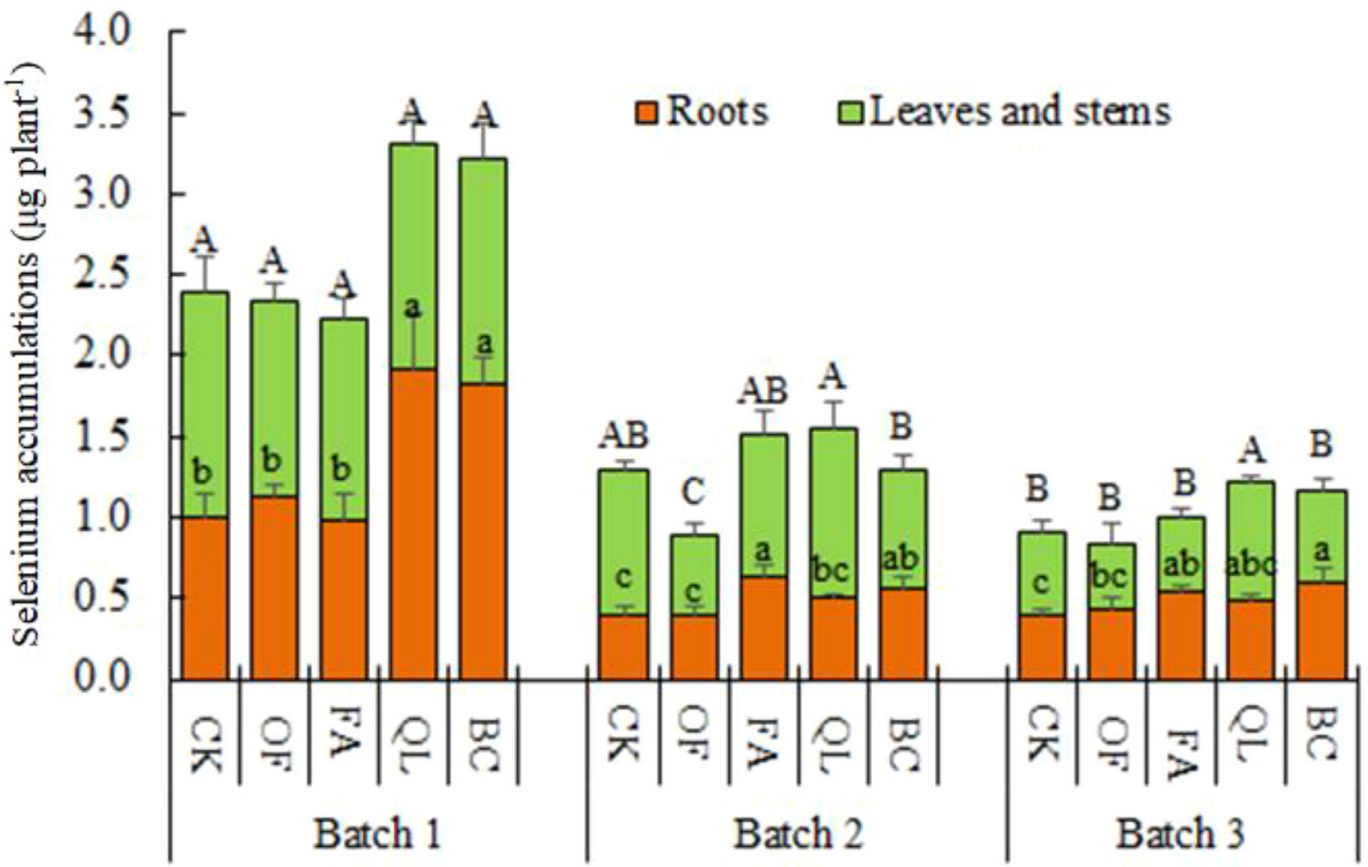
Selenium accumulation in maize under different soil conditioning treatments. Different lowercase letters within the same batch column indicate significant differences in root selenium accumulation (*P* < 0.05); different uppercase letters indicate significant differences in selenium accumulation in stems and leaves (*P* < 0.05).

Selenium accumulation patterns across consecutive maize batches are shown in **Figure 4**. After the first harvest, conditioner treatments significantly affected selenium accumulation in roots but not in stems and leaves. Lime and biochar produced significantly higher root selenium accumulation than potassium humate, organic fertilizer, and CK, resulting in overall increases in total plant selenium accumulation of 38.17% and 35.03%, respectively, relative to CK.

By the second batch, soil conditioners significantly influenced selenium accumulation in both roots and stems/leaves. Root selenium accumulation followed the order potassium humate > biochar > lime > organic fertilizer > CK. Compared to CK, selenium accumulation increased by 58.25%, 39.04%, 27.62%, and 0.49% in the potassium humate, biochar, lime, and organic fertilizer treatments, respectively, with significant increases under potassium humate and biochar. Stem and leaf selenium accumulation ranked as lime > CK > potassium humate > biochar > organic fertilizer, with the organic fertilizer treatment significantly lower than CK. Total plant selenium accumulation increased by 19.02% under lime and 15.97% under potassium humate, while biochar and organic fertilizer decreased accumulation by 1.28% and 32.36%, respectively.

In the third batch, biochar produced the highest root selenium accumulation, followed by potassium humate; both significantly exceeded CK. Lime and organic fertilizer also increased root selenium accumulation, although not significantly. In stems and leaves, lime again showed the strongest effect, significantly surpassing CK and all other treatments, whereas the remaining treatments showed no significant differences from CK. Total plant selenium accumulation followed the order lime > biochar > potassium humate > CK > organic fertilizer. Relative to CK, lime, biochar, and potassium humate increased selenium accumulation by 32.75%, 26.61%, and 9.84%, respectively, whereas organic fertilizer reduced it by 7.62%.

#### 3.4.3 Effects of soil conditioners on the selenium transfer coefficient in maize

Nutrient uptake in plants typically proceeds from the roots to aboveground tissues, and the transfer coefficient reflects the efficiency with which absorbed nutrients are translocated internally. **Table 3** summarizes the selenium transfer coefficient from roots to shoots under different soil conditioner treatments. Both the type of conditioner and the time elapsed after application markedly influenced selenium translocation.

**Table 3:** Selenium transfer coefficient (TI) in maize plants under different treatments.

Treatments	Batch 1	Batch 2	Batch 3
CK	0.3223 ± 0.0125 a	0.4353 ± 0.0275 a	0.1934 ± 0.0149 b
OF	0.2496 ± 0.0042 b	0.2033 ± 0.0360 c	0.1328 ± 0.0100 c
FA	0.3074 ± 0.0363 a	0.2643 ± 0.0268 bc	0.1482 ± 0.0146 c
QL	0.2573 ± 0.0311 b	0.4283 ± 0.0762 a	0.2587 ± 0.0160 a
BC	0.2230 ± 0.0120 b	0.2906 ± 0.0052 b	0.1539 ± 0.0106 c

TI, Transport Index = selenium content in stems and leaves/selenium content in roots. Different uppercase letters within the same batch indicate significant differences among treatments (*P* < 0.05).

In the first maize batch, all soil conditioners reduced the selenium transfer coefficient, with organic fertilizer, lime, and biochar causing the largest declines. By the second batch, the transfer coefficients under all conditioner treatments remained lower than CK, yet lime showed a pronounced increase, becoming statistically indistinguishable from CK and significantly higher than the other conditioners. In the third batch, lime exhibited a significantly higher selenium transfer coefficient than CK, whereas CK exceeded biochar, potassium humate, and organic fertilizer.

Across application cycles, the transfer coefficients under organic fertilizer and potassium humate consistently remained below CK and declined further over time, with organic fertilizer showing the steepest decrease. Biochar also consistently reduced the transfer coefficient relative to CK but displayed a fluctuating pattern—first decreasing, then increasing, and finally decreasing again—yielding values closer to CK than those observed under organic fertilizer or potassium humate. Lime reduced the transfer coefficient only in the first batch but exceeded CK thereafter, indicating that lime ultimately enhances selenium movement from roots to aboveground tissues over longer timescales.

### 3.5 Interrelationships among factors influencing selenium absorption and translocation under different soil conditioners

Correlation analysis between maize selenium nutritional indicators and key soil physicochemical properties and selenium speciation is presented in **Table 4**. Among selenium fractions, soluble selenium showed significant or highly significant positive correlations with selenium content in stems and leaves, selenium accumulation in stems and leaves, and the selenium transfer coefficient. Iron–manganese–bound selenium exhibited highly significant positive correlations with root selenium content, stem and leaf selenium content, selenium accumulation in both roots and shoots, total plant selenium accumulation, and the selenium transfer coefficient. In contrast, residual selenium was highly negatively correlated with all of these indicators. Exchangeable and organically bound selenium showed no significant correlations with maize selenium nutritional parameters.

**Table 4:** Linear correlation coefficients between soil properties, soil selenium fractions, and maize selenium nutritional indicators.

	Se content in roots	Se content in leaves and stems	Se accumulation in roots	Se accumulation in straws	Total Se accumulation in plants	Transport indexes
Soluble- Se	0.172	0.350^**^	0.153	0.299^*^	0.228	0.431^**^
Exchangeable- Se	-0.098	-0.186	-0.130	-0.242	-0.188	-0.181
Fe/Mn oxide-bound-Se	0.565^**^	0.707^**^	0.616^**^	0.797^**^	0.732^**^	0.468^**^
Organic Matter-bound-Se	0.060	0.139	0.051	0.147	0.097	0.173
Residual-Se	-0.470^**^	-0.667^**^	-0.462^**^	-0.687^**^	-0.590^**^	-0.559^**^
pH	0.504^**^	0.633^**^	0.324^*^	0.380^**^	0.367^**^	0.411^**^
Available P	0.203	0.173	-0.007	-0.131	-0.063	-0.056
Available S	-0.032	-0.162	0.029	-0.073	-0.016	-0.290^*^

*indicates *P* < 0.05, **indicates *P* < 0.01.

Regarding soil properties, soil pH displayed significant or highly significant positive correlations with root selenium content, stem and leaf selenium content, selenium accumulation in roots and shoots, total selenium accumulation, and the selenium transfer coefficient. Among available nutrients, available sulfur showed a significant negative correlation only with the selenium transfer coefficient, whereas available phosphorus displayed no significant associations.

Overall, these findings indicate that soil pH is a key environmental factor regulating maize selenium nutrition under soil conditioner treatments. Soluble selenium, iron–manganese–bound selenium, and residual selenium serve as critical indicators of selenium uptake and accumulation, while available sulfur significantly influences selenium translocation within the plant.

**Figure 5:**
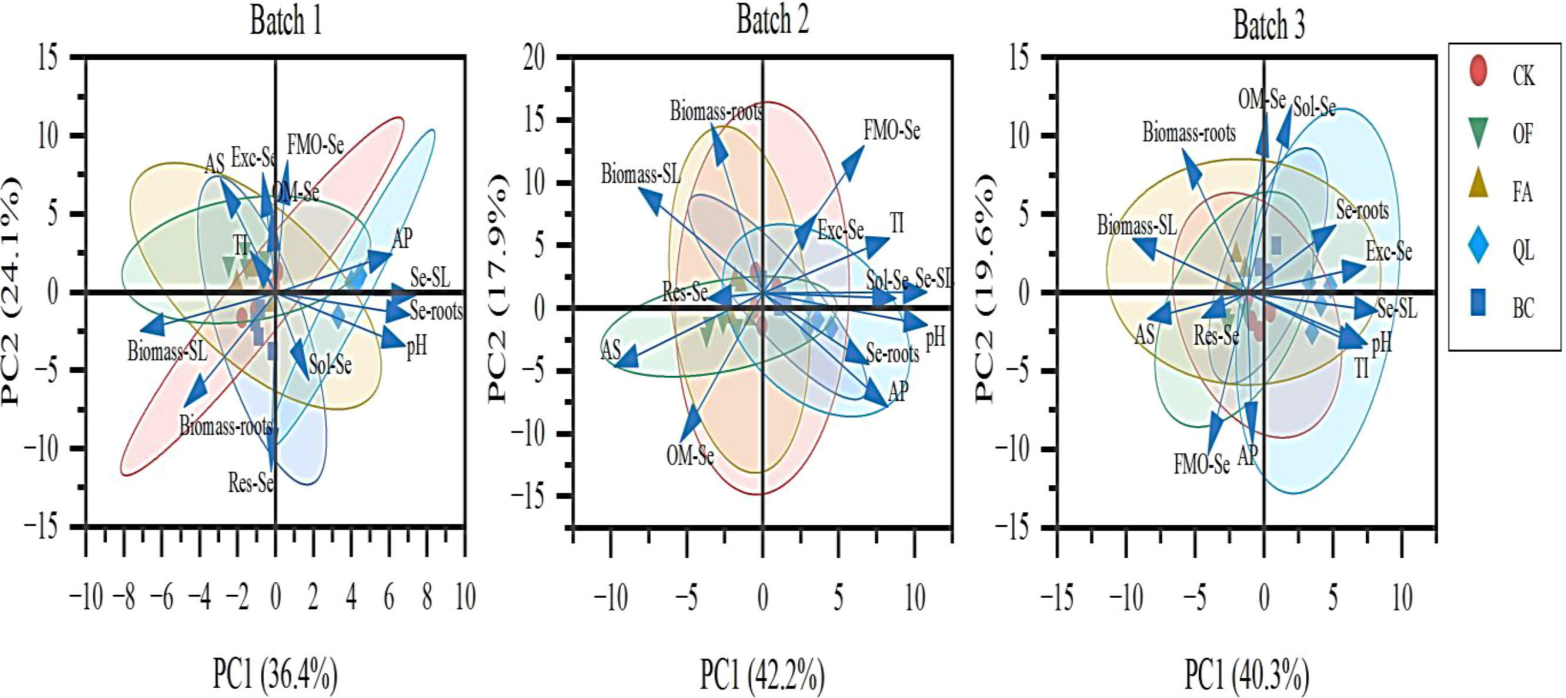
Principal Component Analysis of soil selenium speciation, maize biomass, and selenium yield under soil conditioner addition. CK, OF, FA, QL, and BC represent control, organic fertilizer, potassium humate, lime, and biochar pretreatment, respectively. Sol-Se, Exc-Se, FMO-Se, OM-Se, and Res-Se represent Soluble Se, Exchangeable Fe/Mn oxide-bound Se, Organic matter-bond Se, and Residual Se, respectively. AP and AS represent available phosphorus and available sulfur, respectively. Biomass-roots and Biomass-SL represent the biomass of maize roots and stems/leafs, respectively. Se-roots and Se-SL represent the selenium content of maize roots and stems/leafs, respectively. TI represents the selenium transfer coefficient.

**Figure 5** shows principal component analysis (PCA) of the three batches of maize data with added soil conditioner. The results show that the first two principal components (PC1 and PC2) cumulatively explained 60.5%, 60.1%, and 59.9% of the total variance, respectively, indicating that these two dimensions can reflect the original structure of the data well. In the first batch of maize, the lime application treatment showed a significant difference from the control (CK) treatment. Soil pH, available phosphorus in the soil, and selenium content in different parts of the maize plant were closely related to lime application. In the second and third batches of maize planting, there was significant overlap among the treatments, indicating a weakening of the treatment effect. In the second batch of maize, soluble selenium content, transfer coefficient, selenium content in maize stems and leaves, and soil pH were closely related; selenium content in maize roots was closely related to available phosphorus in the soil. In the third batch of maize, the selenium content in both maize roots and stems and leaves was closely related to exchangeable selenium, while the selenium content in maize stems and leaves, soil pH, and selenium transfer coefficient were closely related.

## 4 Discussion

### 4.1 Effects of soil conditioners on soil selenium species

This study continuously monitored shifts in soil selenium speciation across three sequential maize seedling harvests and found substantial variation in several selenium fractions among batches under the same treatment, particularly in iron–manganese-bound and residual selenium (**Table 1**). Soil pH, available phosphorus, and available sulfur are key factors influencing soil selenium speciation and crop selenium uptake. These fluctuations were likely driven not solely by the applied soil conditioners but also by interacting climatic, hydrologic, and crop-management factors. Research on arsenic speciation indicates that arsenic is initially adsorbed onto iron–aluminum clay mineral surfaces and, as soil pH fluctuates, these minerals transform and incorporate the adsorbed arsenic into their lattices, resulting in more stable fixation (Ran, 2023). Given the structural and chemical similarities between selenium and arsenic, iron–manganese-bound and residual selenium likely undergo comparable inter-conversion during clay-mineral transformations under variable environmental conditions.

Soil conditioners significantly influenced exchangeable selenium after all three maize harvests; soluble selenium after the second and third harvests; residual selenium during the first and third harvests; and organically bound selenium after the second harvest (**Table 1**). Soluble and exchangeable selenium represent the bioavailable fractions in soil, and their availability is strongly regulated by soil pH and organic matter content (Liu et al., 2015). The addition of soil conditioners markedly altered soil pH (**Figure 1**), with treatments containing organic fertilizer or potassium humate producing the lowest pH values and correspondingly the lowest available selenium.

The four soil conditioners used in this study differed substantially in organic-matter characteristics, which influenced selenium sorption and stabilization. Because selenium fixation by organic matter requires time to develop, significant differences in organically bound selenium among treatments did not emerge until the second harvest. Organic fertilizer and potassium humate, both rich in active organic components, produced higher levels of organically bound selenium at that stage. Biochar, characterized by a more stable carbon matrix, exerted a delayed effect, displaying elevated organically bound selenium only after the third maize harvest.

The observed effects of soil conditioners on residual selenium were consistent with previous findings (Tan et al., 2024; Lu et al., 2023). Residual selenium, immobilized within mineral lattices, is largely inaccessible under stable natural conditions. During the first planting cycle, the combined initial activation from irrigation, maize cultivation, and soil-conditioner application likely disrupted lattice structures, leading to treatment-specific alterations in residual selenium. The pronounced differences among conditioners consequently produced varying degrees of influence on this most recalcitrant selenium fraction.

### 4.2 Effects of soil conditioners on selenium absorption and accumulation in maize

The application of soil conditioners increased selenium concentrations in maize roots, though the magnitude of this increase varied markedly among treatments (**Figure 3**). This pattern is consistent with previous findings. For example, Huang et al. (2019) reported that selenium uptake by rice roots differed substantially depending on how sodium selenite was combined with various organic materials, while Ma et al. (2023) found that biochar application in dry-land systems enhanced plant selenium absorption. These differences arise because soil conditioners modify soil physicochemical properties and selenium speciation, influencing plant uptake of distinct selenium chemical forms (Dinh et al., 2019).

Lime application significantly elevated root selenium content, largely due to its strong alkalizing effect on soil pH (**Figure 1**). Soil pH showed a highly significant positive correlation with selenium content in both roots and leaves (**Table 4**), consistent with earlier studies (Lu et al., 2023). Although all conditioners increased root selenium, the selenium content in the aboveground tissues of maize treated with organic fertilizer, potassium humate, or biochar was lower than that of the control (**Figure 3**). This suggests that selenium absorbed in these treatments was not effectively translocated from the roots to shoots. Excessive selenium (Se) acts as a pro-oxidant in plants, causing significant toxicity (selenosis) characterized by stunted growth, root inhibition, chlorosis, and necrosis (Gu et al., 2025). It causes damage by replacing sulfur in amino acids (cysteine and methionine), resulting in dysfunctional proteins, alongside inducing severe oxidative stress (Cao et al., 2025).

Crops can absorb all available selenium species in soil, including Se(IV), Se(VI), and dissolved organic selenium. However, selenium must undergo intracellular transformation before being transported to aboveground tissues. Se(IV) taken up by roots is quickly converted into organoselenium compounds primarily selenomethionine and methylselenocysteine most of which remain in the roots, with only limited translocation to stems (Winkel et al., 2015). In contrast, selenate (Se(VI)) is readily transported through the xylem to aboveground tissues, where it is reduced to Se(IV); following reduction, selenium is incorporated into organoselenium forms and redistributed to various tissues through both xylem and phloem pathways [32]. Transport efficiencies differ widely across chemical forms, with the general order Se(VI) > SeMet > Se(IV)/SeCys (Kikkert and Berkelaar, 2013).

Different soil selenium fractions also contain distinct chemical species (Qu et al., 1997). Soluble selenium is dominated by SeO_4_²^-^, with smaller proportions of SeO_3_²^-^and soluble organic selenium, whereas exchangeable selenium exists mainly as SeO_3_²^-^(Gissel et al., 1984; White, 2016). In this study, soil conditioners significantly affected exchangeable selenium (**Table 1**), and selenium content and transfer coefficients in stems and leaves were significantly positively correlated with soluble selenium (**Table 4**). This indicates that the application of soil conditioners on dryland may mainly affect the content of Se(IV) in available selenium in the soil, thereby affecting the absorption and accumulation of selenium by crops.

Furthermore, plant absorption of selenium involves complex synergistic and competitive relationships with other nutrients. Generally, Se(IV) in the soil is absorbed by phosphorus transport proteins (Zhang et al., 2014), while Se(VI) mainly enters the plant root system through sulfur transport proteins (Gupta and Gupta, 2017). Therefore, the availability of phosphorus and sulfur in the soil plays an important regulatory role in the efficiency of selenium translocation, absorption, and accumulation in plants. Experiments have shown that the combined application of phosphorus and Se(IV) significantly reduces selenium translocation and accumulation in wheat, but the combined application of phosphorus and Se(VI) increases selenium absorption and accumulation in wheat (Zhang et al., 2017). The effect of sulfur on plant selenium absorption and accumulation also varies depending on the crop’s sulfur status (Stroud et al., 2010). The addition of soil conditioners also significantly affects the transformation characteristics of available phosphorus, available sulfur, and selenium forms in the soil, thereby affecting the absorption and translocation of selenium by maize plants. However, as mentioned above, we regret that we did not detect changes in soil Se(IV) and Se(VI). Therefore, we believe that future related studies must measure changes in soil Se(IV) and Se(VI) content to help understand the mechanism of selenium transfer and transformation in soil and plants.

## 5 Conclusions

Different soil conditioners had significant effects on soil selenium speciation. Biochar consistently increased both soluble and exchangeable selenium, whereas lime enhanced soluble selenium but significantly reduced exchangeable selenium after the second harvest compared with the control. All conditioners increased root selenium concentrations, with lime and biochar producing the most pronounced effects. However, except for lime, treatments reduced aboveground selenium content relative to the control, with organic fertilizer and potassium humate causing the greatest declines. Transfer coefficients in all conditioner treatments were lower than those of the control, reflecting their substantial influence on residual selenium. Correlation analysis showed that root selenium, aboveground selenium, and transfer coefficients were all significantly positively correlated with soil pH but not with exchangeable selenium. Soluble selenium content and transfer coefficients were also significantly positively correlated with soil soluble selenium. Overall, applying soil conditioners to selenium-rich dry-land soils increased soil pH and enhanced available selenium, thereby improving selenium uptake by maize roots. However, the selenium forms enhanced by conditioners were less efficiently transported to aboveground tissues. Among the treatments, lime was the most effective at increasing crop selenium content, while biochar was most effective at enhancing soil available selenium. The following aspects should be emphasized the limitations of the study and future studies: Techniques are necessary to develop and utilize such as DGT to improve the accuracy of the evaluation of Se bioavailability. Studies are required to acquire understanding about antagonistic and competitive effects of each nutrient elements (N, P, and S) with Se on plant uptake of Se. Studies are needed to explore the role, as well as the strength of each component (e.g., LMWOAs, HA, FA) in organic materials on Se bioavailability. Models are needed to predict the effects of climate change on the soil distribution of available Se on a large-scale.

## Data Availability

The original contributions presented in the study are included in the article/supplementary material. Further inquiries can be directed to the corresponding authors.
